# ‘TIL DEATH DO US PART? DECLINING WIDOWHOOD AND RISING GRAY DIVORCE, 1980-2017

**DOI:** 10.1093/geroni/igz038.2966

**Published:** 2019-11-08

**Authors:** Susan L Brown, I-Fen Lin

**Affiliations:** Bowling Green State University, Bowling Green, Ohio, United States

## Abstract

Roughly one-third of dissolutions among married persons aged 50 and older occur through divorce rather than widowhood, reflecting the rising gray divorce rate and lengthening life expectancies. We use data from the 1980 Vital Statistics and the 2017 American Community Survey (ACS) to estimate the divorce and widowhood rates among married individuals (aged 50+) in 1980 and 2017 to track how much the widowhood rate has declined and the divorce rate has risen. In 1980, women’s widowhood rates exceeded their divorce rates at all ages. For men, the rate of divorce outpaced the rate of widowhood through ages 50-54. By 2017, divorce rates were higher for women through ages 55-59 and for men through ages 60-64, coinciding with the growth in gray divorce. We also examine subgroup variation in the 2017 patterns and the sociodemographic correlates of having experienced divorce versus widowhood during the past year using the ACS data.

